# Retrospective study 2005–2015 of all cases of fetal death occurred at ≥23 gestational weeks, in Friuli Venezia Giulia, Italy

**DOI:** 10.1186/s12884-020-03074-9

**Published:** 2020-07-01

**Authors:** Lorenzo Monasta, Manuela Giangreco, Emanuele Ancona, Fabio Barbone, Elisa Bet, Pierino Boschian-Bailo, Giovanna Cacciaguerra, Angelo Cagnacci, Melania Canton, Maddalena Casarotto, Manola Comar, Simona Contardo, Michela De Agostini, Francesco De Seta, Giovanni Del Ben, Carla Di Loreto, Lorenza Driul, Stefano Facchin, Roberta Giornelli, Annalisa Ianni, Santo La Valle, Ambrogio Pietro Londero, Marciano Manfè, Gianpaolo Maso, Raffaela Mugittu, Monica Olivuzzi, Maria Orsaria, Vanna Pecile, Roberta Pinzano, Francesco Pirrone, Mariachiara Quadrifoglio, Giuseppe Ricci, Luca Ronfani, Tiziana Salviato, Elisa Sandrigo, Silvia Smiroldo, Alice Sorz, Tamara Stampalija, Marianela Urriza, Michele Vanin, Giuseppina Verardi, Salvatore Alberico

**Affiliations:** 1 Institute for Maternal and Child Health – IRCCS Burlo Garofolo, Trieste, Italy; 2 SOC Ostetricia e Ginecologia, Policlinico S. Giorgio S.p.A, Pordenone, Italy; 3 SC Ostetricia e Ginecologia Pordenone, Azienda per l’Assistenza Sanitaria N. 5 – Friuli Occidentale, Pordenone, Italy; 4 SC Ostetricia e Ginecologia Gorizia – Monfalcone, Azienda per l’Assistenza Sanitaria N. 2 – Bassa Friulana-Isontina, Gorizia, Italy; 5 SC Ostetricia e Ginecologia Palmanova – Latisana, Azienda per l’Assistenza Sanitaria N. 2 – Bassa Friulana-Isontina, Gorizia, Italy; 6 Azienda Sanitaria Universitaria Integrata di Udine, Udine, Italy; 7 Dipartimento di Area Medica, Università degli Studi di Udine, Udine, Italy; 8 Department of Medical, Surgical and Health Sciences, University of Trieste, Trieste, Italy; 9 SC Ostetricia e Ginecologia San Vito – Spilimbergo, Azienda per l’Assistenza Sanitaria N. 5 – Friuli Occidentale, Pordenone, Italy; 10 SOC Ostetricia e Ginecologia San Daniele – Tolmezzo, Azienda per l’Assistenza Sanitaria N. 3 – Alto Friuli-Collinare-Medio Friuli, Gemona del Friuli, Udine, Italy; 11 Università di Modena e Reggio Emilia, Modena, Italy

**Keywords:** Intrauterine death, Stillbirth, Small for gestational age

## Abstract

**Background:**

Intrauterine fetal death (IUFD) is a tragic event and, despite efforts to reduce rates, its incidence remains difficult to reduce. The objective of the present study was to examine the etiological factors that contribute to the main causes and conditions associated with IUFD, over an 11-year period in a region of North-East Italy (Friuli Venezia Giulia) for which reliable data in available.

**Methods:**

Retrospective analysis of all 278 IUFD cases occurred between 2005 and 2015 in pregnancies with gestational age ≥ 23 weeks.

**Results:**

The incidence of IUFD was 2.8‰ live births. Of these, 30% were small for gestational age (SGA), with immigrant women being significantly over-represented. The share of SGA reached 35% in cases in which a maternal of fetal pathological condition was present, and dropped to 28% in the absence of associated pathology. In 78 pregnancies (28%) no pathology was recorded that could justify IUFD. Of all IUFDs, 11% occurred during labor, and 72% occurred at a gestational age above 30 weeks.

**Conclusion:**

The percentage of IUFD cases for which no possible cause can be identified is quite high. Only the adoption of evidence-based diagnostic protocols, with integrated immunologic, genetic and pathologic examinations, can help reduce this diagnostic gap, contributing to the prevention of future IUFDs.

## Background

The intrauterine fetal demise is, for a woman and for a couple, always an enormous psycho-affective trauma [1]. The search for the cause is not only a due act, but is fundamental to improve care by acting on prevention measures.

Fetal death still presents several aspects that have not been ascertained and on which agreement has not been reached. Among these, is the definition of the reference population, in terms of gestational age at the event and fetal weight category [2–7].

In Italy, for many years, the cut-off date that distinguished abortion from fetal death was set at 180 days (25 weeks and 5 days), arbitrarily interpreting Law No. 194/78, which identified this gestational age as the upper limit for voluntary interruption of pregnancy according to Article 6 [8]. This period was considered, at the time of the promulgation of the Law in 1978, the boundary between impossibility and possibility of neonatal survival. Medical evolution has, however, lowered this limit, in some cases permitting neonatal survivals at 23 weeks of gestation. For this reason, the ≥23 weeks limit is frequently employed for the definition of IUFD in the majority of Italian studies [9–11].

This heterogeneity in the definition of fetal death or “stillbirth” makes it difficult to compare incidences in the different populations observed and to accurately assess the extent of the problem.

Every year worldwide there are about 2.6 million IUFD cases at or above 28 gestational weeks, [3] with an incidence ranging from 3.4 per thousand (‰) total births in high-income countries to 36 in the Sub-Saharan and Southern Asia regions [12].

Another obstacle to the accurate evaluation of this phenomenon and the comparison between studies, is the difficulty to identify the underlying cause, which can obviously be ascribed to one of three factors: maternal, placental and fetal. These factors very often interact with one another, leaving space for subjective interpretation when attempting to identify the actual etiology. Some 81 different classification systems for the causes of fetal death have been counted in the literature [13].

From the clinical point of view, regardless of the accuracy of the investigations carried out in IUFD cases, in a quota ranging between 25 and 60% it is impossible to identify a specific pathological condition, in particular if the birth weight centile is unknown [14]. In this regard, the work of J. Gardosi has been crucial: he suggested the adoption of a classification system, named ReCoDe, that classifies cases according to the birth weight centile, corrected for ethnicity, to identify true cases of Small for Gestational Age [15–17]. This allowed growth restriction to be recognized as a cause of IUFD in the absence of any other pathology, even if the blood outflow into the maternal circulation and the post-mortem maceration processes could lead to an overestimation of SGA fetuses.

Finally, the cause of death is often confused with the etiopathogenetic mechanism that determined it. An interesting attempt, aimed at solving this issue, was implemented by Korteweg and colleagues, who proposed a system, adopted prospectively in the Netherlands and named “The Tulip Classification”, [18] designed to identify the cause of IUFD from the pathophysiological mechanism that triggered the chain of irreversible events leading to fetal death. According to the Authors, the adoption of such a classification would allow reducing the cases of unexplained IUFD, obtaining well-defined diagnoses as a result of the full sharing of case information among professionals called to identify them.

Our study aimed to determine the incidence of IUFD over an 11-year observational period in the Italian Region of Friuli Venezia Giulia, and to report on the causes. The secondary aim was to identify the problems both in the causes (i.e. avoidable) and in the failure in their identification, in order to define a shared multidisciplinary diagnostic protocol (obstetric / anatomopathological / genetic / microbiological) to be applied in the regional area, based on actual evidence.

## Methods

This is a retrospective study that involved all of the existing eleven maternity centers of the Friuli Venezia Giulia Region, located in North-East Italy. Friuli Venezia Giulia has 1.2 million inhabitants, and is divided into four provinces. Two of the eleven maternities are tertiary centers, both reaching approximately 1.5 thousand deliveries per year. They are located in Trieste and Udine, the two main towns, of approximately 200 thousand and 100 thousand inhabitants, respectively. The other nine maternities are secondary centers, located both in urban (two in Pordenone, and one in Gorizia and Monfalcone) and rural areas (Tolmezzo, San Vito al Tagliamento, Latisana, San Daniele, Palmanova).

We identified all intrauterine fetal deaths (IUFDs) occurred ≥23 gestational weeks, for the years 2005–2015, by event, date of event and birth center. We were unable to guarantee the quality of the data recorded prior to 2005. The choice of the 23rd gestational week cut-off was made based on the reasons mentioned in the Introduction section. IUFDs were identified through the hospital discharge records (SDO: *Scheda di Dimissione Ospedaliera*) data system. Available information on the number of events and the dates were then sent to all birth centers. The obstetricians designated for the study were asked to complete an electronic questionnaire recovering information from medical records. The questionnaire was developed by the study coordinator (SA) with the support of the epidemiology unit of the coordinating center (Clinical Epidemiology and Public Health Research Unit of the Institute for Maternal and Child Health – IRCCS Burlo Garofolo, Trieste, Italy), designed to obtain information on the epidemiological characteristics of the women whose pregnancies ended with an IUFD, and to identify specific risk factors attributable to the IUFD, relating to conditions either preceding conception or occurring during gestation. The classification adopted was based on similar classifications available in the literature, [19] and in particular on the NICE Guideline 121, [20] modified according to the definition of obstetric pre-post conception risk factors adopted by the Centre for the management of at-risk pregnancies of the Institute for Maternal and Child Health – IRCCS Burlo Garofolo.

Fetal growth restriction (FGR) was reported among the fetal conditions, and was considered as an anomaly in growth parameters at ultrasound during gestation. At delivery, in most cases, fetuses were also classified by their weight by gestational age, and were classified as small for gestational age if their weight was below the 10th centile of the normal birth weight distribution.

Each center then gathered and sent back to the coordinating center the information for analysis.

Statistical analyses were mainly descriptive. Data are presented as frequencies and percentages, and medians with interquartile ranges (IQR). *P*-values for the significance of associations in 2 × 2 tables were the result of two-tailed Fisher exact tests. For the comparison of continuous variables between two groups, we carried out Mann-Whitney rank-sum tests. A *p*-value< 0.05 was considered significant. To analyze the time trend in the number of IUFDs per year, we calculated weighted moving averages, adding the crude value for the reference year with the previous and next weighted 0.5, and dividing by 2. The first and last values were calculated adding the crude value for the reference year with the next, or previous, respectively, weighted 0.5, and the sum was divided by 1.5. All analyses were carried out with SAS 9.4 (SAS Institute Inc., Cary, USA) and Stata/IC 14.2 (StataCorp LLC, College Station, USA).

## Results

For the period 2005–2015, 303 IUFD cases were identified through the electronic record system. These represent an incidence of 2.8‰ over the number of live births in the same time period (*n* = 108,351 according to the Microdata Repository of Friuli Venezia Giulia), with a substantially constant trend in the considered years. This incidence does not take into account the voluntary termination of pregnancies for fetal malformations before the 23rd week of gestation. Fetal malformations among the IUFDs identified were the consequence of the woman’s choice not to terminate the pregnancy, or of missed detection in the prenatal period.

**Fig. 1:**
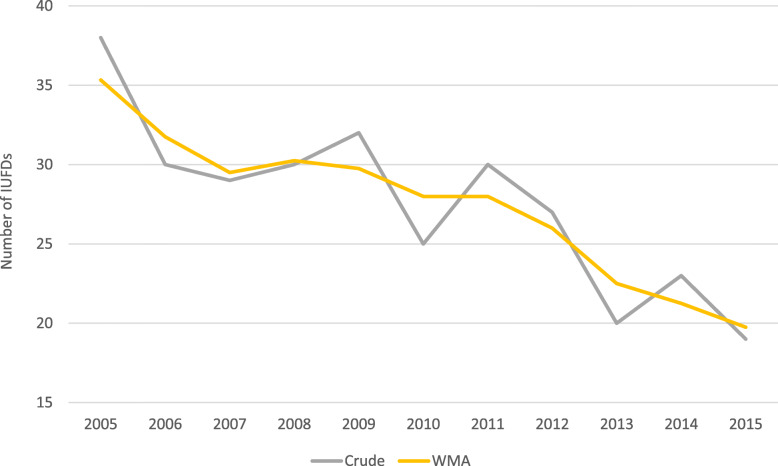
Time distribution of cases of intrauterine fetal deaths (IUFDs) (grey line) and corresponding weighted moving average (WMA, in yellow)

The analysis of the time trend of these 303 IUFDs, adopting a weighted moving average, shows a downward trend from 2005 to 2015, with the actual number going from 38 in 2005 to 19 in 2015 (Fig. 1). The rate decreased from 3.1‰ in the period 2005–2008, to 2.8‰ in 2009–2012, to 2.3 ‰ in the period 2013–2015.

Of these 303, however, only 278 medical records could be identified by the maternity centers and were thus analyzed in our study. The characteristics of the 25 missing IUFD were almost impossible to analyze, as we could only count on the date of event and the maternity center where it occurred. Centers had from 0 to 29% missing records and, throughout the years, we went from 0 missing in 2010 and 2013 to 27% in 2006.

**Fig. 2:**
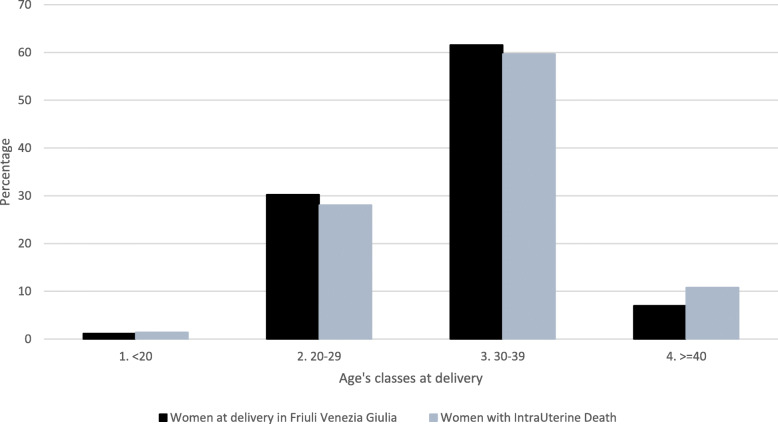
Women who suffered an intrauterine death at ≥23 weeks of gestation, compared to women with live-birth deliveries, by age group: period 2005–2015

The population of women who experienced IUFD was divided into four age groups and is shown in Fig. 2, together with a comparison of the same age distribution of women at delivery (live births).

We did not find significant differences in the distribution of age of women suffering IUFDs compared to women with live birth deliveries. About 60% of IUFD cases occurred in the 30–39 age groups, with a median age of 33 years (IQR 29–36), if compared to women with a live-birth delivery (median age 32 years: IQR 28–36; *p* = 0.14). The percentage of primigravidae was 59%, which is line with population of women delivering a live-born child in the same Region.

Table 1 shows the proportion of women suffering IUFD compared to women with live-birth deliveries in Friuli Venezia Giulia region, by ethnic origin. Significant differences, with higher IUFDs, were registered for women of African (two-tailed Fisher exact test: *p* = 0.008) and Latin American origin (*p* = 0.005).

**Table 1:** Women who suffered an intrauterine death at ≥23 weeks of gestation, compared to women with live-birth deliveries, by ethnic origin: period 2005–2015

Origin	Caucasian N (%)	African N (%)	Asiatic N (%)	Latin American N (%)	Total N (%)
Women with Intrauterine Death	241 (87%)	24 (9%)	5 (2%)	8 (3%)	278 (100%)
Women with live-birth deliveries	93,770 (91%)	5036 (5%)	3015 (3%)	969 (1%)	102790^a^ (100%)
*p*-value	**0.011**	**0.008**	0.370	**0.005**	

^a^ Total women were 110,415, but 7625 (6.9%) had missing data on origin

*p*-values are calculated for each category against all other categories together; two-tailed Fisher exact test

**Fig. 3:**
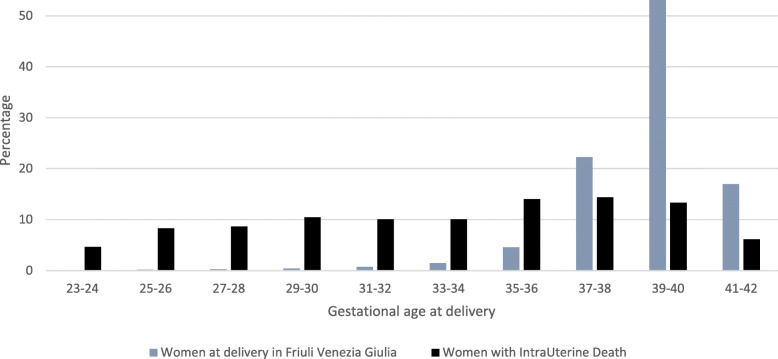
Women who suffered an intrauterine death at ≥23 weeks of gestation, compared to women with live-birth deliveries, by gestational age at event: period 2005–2015 Note to Fig. 3. The rate of IUFD over live births, by gestational age, is the following: Overall: 2.6‰; 23–24 weeks: 120.4‰; 25–26: 125.0‰; 27–28 weeks: 88.6‰; 29–30 weeks: 66.8‰; 31–32 weeks: 35.9‰; 33–34 weeks: 17.8‰; 35–36 weeks: 7.9‰; 37–38 weeks: 1.7‰; 39–40 weeks: 0.6‰; 41–42 weeks: 0.9‰.

The median gestational age at IUFD was 34 weeks (IQR 30–38). Regarding gestational age, the rate of IUFDs over live births decreases dramatically from 120.4‰ in the 23–24 weeks of gestation group to 0.6‰ and 0.9‰ of the 39–40 and 41–42 weeks of gestation groups, respectively (Fig. 3 and note to Fig. 3). Despite this trend, 94 cases (34%) occurred > 36 gestational weeks.

In 54% of the IUFDs, the fetus was a male, compared to 51% of live births (*p* = 0.549).

The percentage of IUFD with birthweight below the 10th centile for the respective sex and gestational week, according to Italian reference growth curves, [21] was 30% (83 over 278).

Out of the 257 IUFD for which we had data, 242 had done a 20 weeks ultrasound screening for fetal congenital malformations. Through this screening, twelve major malformations were identified, four of which also presented minor malformations. In addition, three minor malformations, compatible with life, were also recorded. The overall incidence of fetal malformations among IUFDs was 6%.

In 14 (5%) cases of IUFD, fertilization had been obtained in vitro (IVF), compared to 2% in live-birth deliveries. In four cases (1.4%) the cause of IUFD was identified as a seroconversion for a TORCH complex during gestation.

The presence of a nuchal cord was described at birth in 55 fetuses (20%). Of these, 23 also presented other pathologies, nine did not but were SGA, while 23 did neither have other pathologies nor were SGA. The nuchal cord was, however, never reported as the cause of fetal death.

In the medical records, 162 women had at least one high-risk condition reported (58%) (Tables 2 and 3). In this group, 36% IUFDs were under the 10th centiles of weight (small for gestational age: SGA) (Table 3). If we consider SGA as an additional potential cause of IUFD, even in the absence of other reported conditions, this leaves 79 cases of IUFD (28%) with no attributed risk factor.

**Table 2:** Conditions that could have been associated with the intrauterine death, as reported in the medical records of the women

Class of Pathology	Pathology	N	% over 278 IUFDs
Fetus	Congenital anomalies incompatible with life	7	2.5
	Chronic infections (i.e. TORCH Syndrome)	4	1.4
	Acute infections	21	7.6
	Non-immune fetal hydrops	5	1.8
	Isoimmunization	1	0.4
	Feto-maternal transfusion	0	0.0
	Feto-fetal transfusion syndrome	1	0.4
	Fetal Growth Restriction	43	15.5
Placenta	Placental abruption	36	12.9
Amniotic Fluid	Chorioamnionitis	32	11.5
Uterus	Uterine Rupture	2	0.7
Maternal o Mother	Diabetes	29	10.4
	Essential hypertension	14	5.0
	Pre-eclampsia	27	9.7
	Lupus/Antiphospholipid antibody syndrome	4	1.4
	Cholestasis	5	1.8
	Alcohol abuse	1	0.4
	Smoking Abuse (> 5 cigarettes/day)	6	2.2
	Drug addiction	1	0.4
During delivery	Asphyxia	9	3.2
	**Total conditions reported**	**248**	

The 248 conditions were reported for 162 IUFD cases. In addition, at least another 30 fetuses with no other condition were small for gestational age (SGA), while we did not have information on SGA on other 20 cases. The remaining 79 cases did not present any condition and were not SGA

**Table 3:** Women with reported conditions that could have been associated with the intrauterine death, and evaluation of growth of the fetus at IUFD

	No Small for Gestational Age	Small for Gestational Age	Total	*p*-value
Conditions				
- No condition	79 (72%)	30 (28%)	109 (100%)	
- At least one condition	96 (64%)	53 (36%)	149 (100%)	0.180*
Hypertension in pregnancy				
- No hypertension in pregnancy	156 (70%)	66 (30%)	223 (100%)	
- Hypertension in pregnancy	19 (53%)	17 (47%)	36 (100%)	0.053*
Total	175 (68%)	83 (32%)	258 (100%)**	

* Two-tailed Fisher exact test

** Missing data due to lack of data on sex (recorded as ambiguous) or weight. Of the 20 missing in this table, 13 presented at least one condition

We then evaluated, based on the ultrasound charts, in how many cases a deviation of the biometric parameters of fetal growth was found, indicating a restriction. Intrauterine fetal growth restriction was reported in 43 cases (16%), of which 30 (70%) were considered SGA at birth, as defined by the center.

There were 36 cases (13%) of placental abruption. Of these, eight (22%) occurred during labor and 12 (33%) showed an association with a maternal hypertensive/pre-eclamptic disease.

**Fig. 4:**
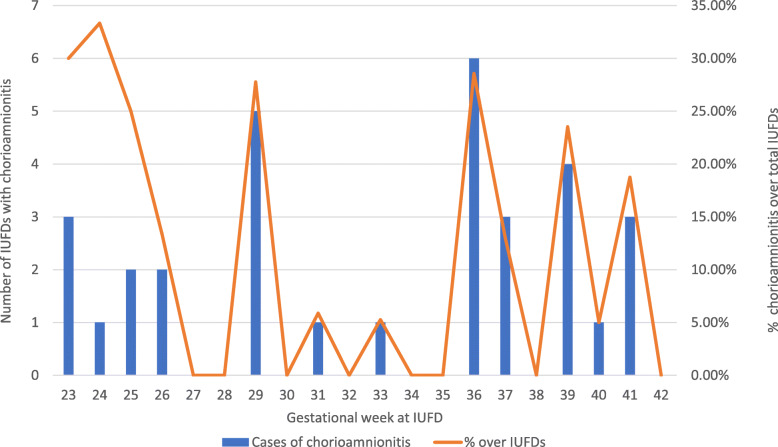
Frequencies and percentages of chorioamnionitis among intrauterine deaths (IUFDs), by gestational week at IUFD

The assessment of the medical records allowed the identification of 32 cases (11%) presenting clinical signs of chorioamnionitis in pregnancy. Given the retrospective nature of the study, we were unfortunately unable to recover information on the full diagnostic path and the simultaneous presence of maternal or fetal vascular malperfusion. In Fig. 4, these cases are stratified by gestational week at IUFD, and we notice that cases are distributed quite evenly throughout the gestational ages considered.

The amniotic fluid assessment in the last performed ultrasound showed the presence of oligohydramnios in 23 cases (8%), with 12 of these (52%) presenting SGA fetuses at delivery. Ten cases (4%) polyhydramnios were also identified, but we did not find associations with diabetes, isoimmunizations, nor other conditions.

Finally, we identified 32 cases (11%) which occurred during labor, which had started spontaneously. These are shown, stratified by gestational age, in Table 4.

**Table 4:** Frequencies and percentages of intrauterine deaths (IUFDs) occurred during labor, by gestational week at IUFD

Gestational week at intrauterine death	IUFDs occurred during labor
23–24	6 (19%)
25–26	0 (0%)
27–28	1 (3%)
29–30	2 (6%)
31–32	1 (3%)
33–34	3 (9%)
35–36	2 (6%)
37–38	6 (19%)
39–40	3 (9%)
41–42	8 (25%)
**Total**	**32 (100%)**

In the diagnostic evaluation of the dead newborn, a photographic documentation was available only in 4% of the cases, and the autoptic examination of the fetus had been performed only in 21% of the cases, even if the histological examination of the placenta, carried out by pathologists often not experts of this field, had been carried out in 95% of cases.

For the 258 cases for which we had information both on conditions associated to IUFD and SGA, there was no difference in the percentage of those in which neither of these conditions was present by comparing fetuses that received an autopsy (17/54, 31%) to those that did not (62/204, 30%; *p* = 0.870).

## Discussion

This study offers several elements for the discussion of unresolved issues related to IUFD cases, which make it difficult, among other things, to assess the phenomenon in the various areas observed, limiting interventions, both on a clinical and health management basis, aimed at reducing its incidence to the minimum.

Our decision to focus on IUFD cases with a gestational age ≥ 23 weeks, regardless of birth weight, depended on the practice of most Italian obstetricians to allow the voluntary termination of pregnancy to be completed up to the 22nd completed week + 6 days, considering the higher probability of having neonatal survivals after this limit, as previously explained. In this way the population we studied was homogeneous, having excluded the majority of pregnancies with malformed fetuses, which in Italy generally undergo a voluntary termination of pregnancy. This also explains the low percentage of IUFDs with malformations detected in this study.

The recorded incidence of IUFD – 2.8‰ live births, and decreasing from 3.1 during the period 2005–2008 to 2.3 in 2013–2015 – is in line with that reported by other studies, carried out in high-income countries. It must be emphasized, however, that in our case, most pregnancies with fetal malformations had been excluded for the reasons explained above [21]. Our reported figure is certainly lower than the 6.2‰ figure reported in the USA in 2004, considering IUFDs occurring from the 20th week of gestation, [22] the 7.1‰ registered in Australia following the WHO criteria, [23] and higher than the one reported in the UK in 2015, of 1.1‰ [24].

With regard to maternal age, the highest IUFD rate, over live birth deliveries, was in the > 40 age group (4.0‰), followed by the < 20 age group (3.1‰). The 20–29 and 30–39 had respectively 2.4‰ and 2.5‰. 60% of all IUFD cases fell into the 30–39 years age group, which corresponds to the percentage of women in the same age group delivering in our Region. In similar studies, the distribution of IUFDs cases in relation to live birth deliveries was quite similar [25].

The distribution of IUFD cases by gestational age is comparable to that of the general population of our Region, [26] in contrast with other studies, that observed a progressive decrease in the prevalence of IUFDs as gestational weeks increase [27].

In analogy with what reported by other authors [6, 28–30] a higher risk of IUFD prevalence emerged in women of African origin (*p* < 0.003) compared to the Caucasians. A greater risk was also observed in women of Latin-American origin (*p* < 0.004). An open question is whether this is due to characteristics of greater risk of death in utero in these ethnic groups, or to the difficulty of taking full advantage of the socio-health conditions of the country in which they are immigrants, welfare conditions, which could have limited the phenomenon. In some studies, the greater prevalence of IUFDs observed in Black women was associated with a state of pregnancy-induced hypertension [28, 29]. Given the small number of women with African origin and the small number of women with hypertension, we could not find a significant association between origin and hypertension (*p*-value = 0.12).

Even though we reported the percentage of nuchal chord in our population (20%), it is important to note that a) this percentage is lower than that found in our records of deliveries, which is around 30%, and b) nuchal chord was never identified as the cause of fetal death.

It is known that fetal growth restriction is a risk factor for fetal death [15–17]. In our study, the prevalence of SGA births was 30%, higher than that normally observed in the general population, which is around 10% by definition. The higher percentage of fetal growth restriction in IUFDs finds solid confirmation in similar studies [9, 10, 17, 31].

The identification of pre-gestational and gestational diseases, including obstetric conditions, as well as those of fetal relevance, also occurring in association, showed that in a proportion of 42% of cases (108/259) there was no pathological, maternal/placental/fetal condition that could justify the adverse outcome verified. By including the condition of SGA as risk factor, this percentage decreased to 30% of the cases (78/259), confirming on the role played by the restriction of fetal growth in the determinism of the IUFD.

In the group of pregnant women in whom a pathology was present, the prevalence of SGA was 36% (53/149). This indicates that a woman having at least a pre-pregnancy or pregnancy disease is at higher risk of having a fetal growth restriction, which is certainly a factor to be always considered for its association with IUFD.

There is no doubt that there may also be unknown pathological factors, in the biochemical, genetic or immunological field, which do not allow for a complete identification of the pathologies leading to IUFD in all cases. Towards these factors we need focus our attention and, above all, our post-natal investigations. In our opinion, however, this gap may also depend on a non-exhaustive and complete diagnostic procedure, applied in these of IUFD after birth.

Consideration must be made on the high frequency of cases in which a condition of chorioamnionitis (11%) had occurred, a share that is certainly higher than the values ​​reported in the literature for this pathology and that is usually estimated in the range of 1–4% [32, 33]. Chorioamnionitis was defined by the association of clinical signs of maternal inflammation with fetal tachycardia. However, the retrospective nature of our study did not always allow us to confirm the reported diagnosis.

We need to admit that the reported diagnostic pathway implemented on the dead newborn was inadequate. Genetic diagnoses were only available during gestation in 16% of cases, not significantly increased after fetal expulsion. A picture of the newborn was only done in 4.4% of cases, and autopsy on the fetus was carried out only in 11% of cases. The application of specific and shared anatomopathological classification systems, implemented both on the fetus and on the placenta, by experienced IUFD specialists, could contribute to the reduction of cases in which no cause could be identified. It is known that the proportion of unexplained IUFDs is reduced in those situations where shared and systematic diagnostic criteria and procedures are applied [34]. We believe this aspect is also important to give a just and dutiful answer to the couple that suffered this dramatic event, which in our population occurred after 30 weeks of gestation in 72% of the cases, when the chances of newborn survival increase significantly with good intensive care, and after 36 weeks in 34% of cases, meaning at term.

Some 88% of IUFD cases recorded in this study occurred in the antepartum period and this figure is in line with what was reported by other works carried out in high-income countries, where 90% of deaths in utero occur precisely in this period and are often associated with preventable risk factors, such as obesity, smoking, failure to identify pregnancies at risk [6, 27–35]. Therefore, being able to understand the cause of death in utero would allow addressing the available resources more appropriately to combat the etiological factors on which it is possible to implement concrete prevention.

The last aspect deserving attention, and which we consider worthy of further evaluation, concerns the 11% share of cases in which fetal death occurred during labor (Table 4). The question to be answered in this regard is whether adequate and correct obstetric management could reduce this burden to the minimum, by implementing quality improvement programs, already realized elsewhere [25].

## Conclusion

The percentage of IUFD cases in which no possible cause could be identified is still quite high. Only the adoption of evidence-based diagnostic protocols, with integrated immunologic, genetic and pathologic examinations, can help reduce this diagnostic gap, contributing to the prevention of future IUFDs.

## Data Availability

The complete set of tables generated in the analysis of the data related to the current study are available from the corresponding author on reasonable request.

## Abbreviations

CDC: U.S. centers for disease control and prevention
IQR: Interquartile range
IUFD: Intrauterine fetal death
IVF: In-vitro fertilization
NCHS: National center for health statistics
SGA: Small for gestational age
WHO: World health organization
